# May vestibular rehabilitation reduce apnea-hypopnea index in patients with obstructive sleep apnea? A pilot study

**DOI:** 10.3389/fneur.2025.1664860

**Published:** 2025-12-01

**Authors:** Annalisa Pace, Giannicola Iannella, Saverio Nicoletti, Paola Di Mauro, Francesca Mattioli, Salvatore Cocuzza, Antonino Maniaci, Danilo Alunni Fegatelli, Annarita Vestri, Giuseppe Magliulo

**Affiliations:** 1 Department of Sensory Organs, Sapienza University of Rome, Rome, Italy; 2 Department of Medical and Surgical Sciences and Advanced Technologies “GF Ingrassia” University of Catania, Catania, Italy; 3 Department of Medicine and Surgery, University of Enna Kore, Enna, Italy; 4 Department of Otorhinolaryngology, University of Catania, Catania, Italy; 5 Department of Life Sciences, Health and Health Professions, Link Campus University, Rome, Italy; 6 Department of Public Health and Infection Diseases, Sapienza University of Rome, Rome, Italy

**Keywords:** vestibular system, obstructive sleep apnea, vestibular rehabilitation, apnea-hypopnea index, Functional Head Impulse Test, polysomnography

## Abstract

**Introduction:**

The vestibular system is essential for maintaining the perception of head orientation and acceleration in all directions. The Functional Head Impulse Test (fHIT) is a novel tool for assessing the vestibulo-ocular reflex (VOR) and forms the basis for a recently developed vestibular rehabilitation system. This pilot study aimed to determine whether vestibular rehabilitation alone could objectively improve clinical parameters in patients with obstructive sleep apnea (OSA).

**Methods:**

Twenty male patients diagnosed with OSA underwent baseline polysomnography (PSG) and fHIT assessment. Participants then completed a 10-day vestibular rehabilitation program using the reHAB system, after which PSG and fHIT were repeated.

**Results:**

Initial fHIT analysis indicated impaired vestibular function in 30% of patients. The Wilcoxon signed-rank test demonstrated a statistically significant reduction in the apneahypopnea index (AHI) following rehabilitation (*p* = 0.003), while the change in oxygen desaturation index (ODI) did not reach statistical significance (*p* = 0.082). Scatter plot analysis revealed a moderate positive correlation between changes in AHI and ODI pre-and post-rehabilitation.

**Discussion:**

These preliminary findings suggest a functional connection between vestibular inputs and sleep–wake pathways, possibly mediated by parabrachial circuits and orexinergic modulation.

## 1 Introduction

The vestibular system plays a critical role in maintaining the perception of head position and acceleration in all directions. This is primarily achieved through the vestibulo-ocular reflex (VOR) and vestibulo-spinal reflexes, which integrate sensory input from the peripheral structures of the inner ear and transmit efferent signals to coordinate eye and body movements (1). To evaluate vestibular function, various diagnostic methods are employed, each targeting specific components of the posterior labyrinth. Among these, the Functional Head Impulse Test (fHIT) is a recently developed tool that assesses VOR function by stimulating all semicircular canals and, indirectly, both the superior and inferior branches of the vestibular nerve (2). The fHIT enables objective measurement of a patient’s capacity to maintain accurate visual fixation during rapid head movements, thereby providing valuable insight into the integrity of vestibular pathways.

Recent evidence suggests a potential role for the vestibular system in the regulation of sleep (3). Anatomically, the vestibular nuclei are located in close proximity to the respiratory centers, including the parabrachial nuclei, and are interconnected with the suprachiasmatic nucleus (SCN) of the hypothalamus via the lateral geniculate area. The SCN is recognized as the principal regulator of the biological circadian rhythm governing sleep and wakefulness (4–6).

In a 2022 study, Pace et al. reported vestibular dysfunction in 68% of patients with obstructive sleep apnea (OSA), as assessed by the fHIT (7).

Building on these findings, the present preliminary study aims to investigate whether vestibular rehabilitation, delivered exclusively via the reHAB system, may objectively improve clinical parameters of OSA in patients without other comorbidities.

## 2 Materials and methods

This prospective study was conducted at the Department of Sensory Organs, Sapienza University of Rome between September 2023 and December 2024.

**Figure 1:**
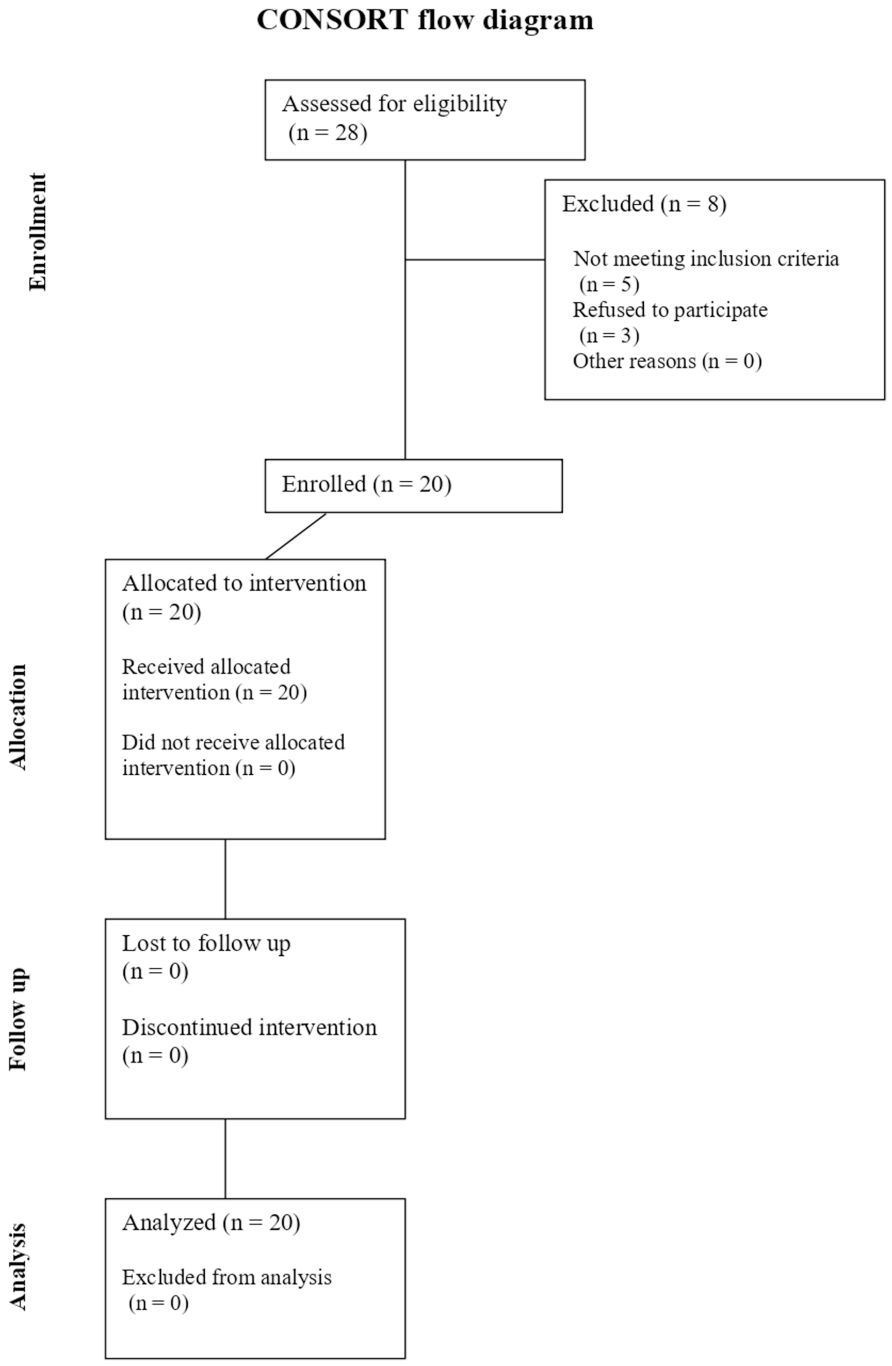
A CONSORT flow diagram is provided to depict the enrollment process, including screening, exclusions, allocation, follow-up, and analysis.

Participants aged over 18 years with a confirmed diagnosis of obstructive sleep apnea (OSA) were enrolled. A CONSORT flow diagram is provided to depict the enrollment process, including screening, exclusions, allocation, follow-up, and analysis (Figure 1). Exclusion criteria included the presence of cardiovascular, metabolic, or neurological diseases; a history of ENT surgical procedures; audiological or vestibular disorders; current imbalance symptoms (Dizziness Handicap Inventory score <40) (8). Patients who had previously undergone domiciliary therapy for OSA (e.g., continuous positive airway pressure, oral appliances, nasal devices) were also excluded.

Participants were restricted to male subjects for this pilot phase. This choice reflected the predominantly male referral pattern during the recruitment period and, at the same time, was intended to limit heterogeneity in order to obtain a clearer estimate of the effect of the vestibular rehabilitation protocol.

Demographic data, including height, weight, body mass index (BMI), and age, were recorded in a dedicated database.

All participants underwent an ENT examination and a one-night type III polysomnography (PSG), in accordance with the 2017 classification criteria of the American Academy of Sleep Medicine (AASM) (9). The PSG device recorded total sleep time, thoracic and abdominal respiratory movements, airflow, heart rate, arterial oxygen saturation, and body position.

The data were also analyzed according to AASM criteria. Apnea was defined as a ≥90% reduction in airflow lasting at least 10 seconds. Hypopnea was identified as a ≥30% reduction in airflow, accompanied by either an arousal or a ≥3% decrease in oxygen saturation, regardless of duration (10–12).

The Apnea-Hypopnea Index (AHI) was calculated as the average number of apneic and hypopneic events per hour of sleep. Two independent reviewers (AP and FM) evaluated all PSG recordings, while a third reviewer (GM) conducted random quality assessments to ensure accuracy and consistency.

For each PSG recording, the following parameters were extracted: AHI (in supine and non-supine positions), oxygen desaturation index (ODI; supine and non-supine), and mean oxygen saturation (SpO₂; supine and non-supine). The average AHI, calculated from both supine and non-supine values, was used as the principal indicator of obstructive sleep apnea (OSA) severity, in accordance with AASM guidelines.

Patients with AHI of less than 5 events per hour, indicative of a healthy condition, were excluded from the study. Patients diagnosed with obstructive sleep apnea (OSA) were stratified into three severity categories based on AHI values: mild OSA (AHI ≥5 and <15), moderate OSA (AHI ≥15 and <30), and severe OSA (AHI ≥30).

All enrolled participants underwent the Functional Head Impulse Test (fHIT) using the BeOn-Solution system. This assessment is based on the patient’s capacity to recognize an optotype displayed on a monitor during rapid, passive head rotations across different angular accelerations (13, 14).

Testing was conducted in a dark room with the subject seated at a distance of 150 cm from a monitor connected to the fHIT device. Prior to the examination, each participant’s visual acuity was verified using distance-scaled, white Landolt C optotypes presented on the screen. Only individuals with a normal visual acuity of 0.2 logMAR or corrected acuity ≤0.5 logMAR were included.

A gyroscope was mounted on the subject's head to measure angular velocity during the test. The fHIT was administered by a trained operator (FM), who applied passive, brief, rotational head impulses along the planes of each semicircular canal pair (left and right horizontal; left anterior-right posterior; right anterior-left posterior). During each impulse, an optotype appeared on a black screen for 80 milliseconds. The patient responded by selecting one of eight possible Landolt C optotypes displayed on a keyboard. For each canal plane, 20 head impulses were administered in both directions.

The fHIT software calculated the percentage of correct answers (%CA) for each impulse series, with values below 80% considered pathological (13, 14).

Vestibular rehabilitation was performed using the reHAB system (BeOn-Solution), a device designed to track and support the recovery of vestibular function over time. The system allows for assessment both before and after a rehabilitation protocol, providing a quantitative evaluation of recovery progress.

The reHAB hardware comprises a tablet for visual stimulus presentation and a gyroscope affixed to the patient’s head to capture angular velocity during exercises. Patients were instructed to move their heads in specific directions according to the targeted semicircular canal, and to identify numbers presented on the screen for 80 milliseconds. A structured exercise protocol was developed to stimulate each semicircular canal, and the Corsi Block-Tapping Test was integrated into the rehabilitation regimen (15).

Participants underwent initial training to familiarize themselves with the rehabilitation program, which they subsequently performed at home for a duration of 10 days. Each patient performed a 10-min session comprising eight exercises approximately 30 min before bedtime, every evening. Adherence was monitored by downloading usage data from the device. Because timing was standardized across participants, the schedule did not influence outcomes. Following this rehabilitation period, the fHIT and polysomnography (PSG) assessments were repeated.

### 2.1 Ethical statement

The study was approved by the local ethical committee (RIF: 6267), following the principles of the Declaration of Helsinki. Each patient enrolled in the study signed informed consent.

### 2.2 Statistical analysis

**Figure 2:**
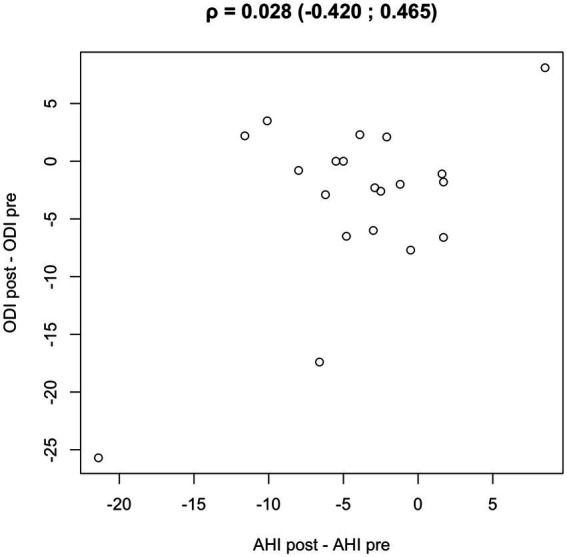
Scatterplot showing the monotonic association between AHI and ODI, with Spearman’s rank correlation coefficient (*ρ*).

Numerical data were summarized as median (IQR). Violin plots, with boxplots overlaid, were used to represent numerical variables. The Wilcoxon signed-rank test assessed differences among groups. Spearman’s rank correlations were used to assess monotonic associations between changes (ΔAHI, ΔODI, ΔT90) (Figure 2).

All the analyses were performed using the statistical software R (version 4.3.0).

## 3 Results

A total of 20 Caucasian male patients diagnosed with OSA were enrolled in the study. Demographic and anthropometric data, including age, weight, height, and body mass index (BMI), are presented in Table 1.

**Table 1:** Patients’ characteristics and PSG values pre and post reHAB.

Pt	Age	Weight (Kg)	Height (cm)	BMI (Kg/m^2^)	Pre reHAB PSG		Post reHAB PSG	
					AHI	ODI	AHI	ODI
1	41	93	179	29	14	13	9.2	6.5
2	49	90	180	28	17	17.8	15.8	15.8
3	46	57	168	24	19.5	6.5	7.9	8.7
4	20	71	183	21	16	15.7	13.5	13.1
5	51	80	173	27	24	26.4	17.4	9.0
6	27	85	182	26	9.5	12.2	6.5	6.2
7	45	83	180	26	16.5	17.3	18.1	16.2
8	52	70	174	23	39	39.4	17.6	13.7
9	43	86	179	27	19.5	11.7	14.0	11.7
10	46	135	188	38	22.6	24.6	24.3	18.0
11	27	125	198	32	24.7	22.1	22.6	24.2
12	42	76	174	25	14.1	17.2	15.8	15.4
13	53	94	151	41	23.9	29.1	20.0	31.4
14	57	64	164	24	20.9	20.8	15.9	20.8
15	50	80	180	25	25.4	21.9	17.4	21.1
16	50	92	182	28	35.2	36.4	43.7	44.5
17	37	94	187	27	32.0	26.8	21.9	30.3
18	40	87	173	29	26.2	28.3	23.3	26.0
19	59	105	178	33	28.6	37.6	28.1	29.9
20	47	72	175	24	22.9	14.9	16.7	12.0
Median (IQR)	46 (41–50)	85 (75–93)	179 (174–182)	27 (25–29)	22.9 (16.8–25.8)	21.35 (15.5–27.1)	17.4 (15.3–22.1)	16.0 (11.9–24.6)

Based on the AASM classification, patients were subgrouped by OSA severity as follows: three patients (15.0%) exhibited mild OSA, 14 patients (70.0%) had moderate OSA, and 3 patients (15.0%) had severe OSA. Apnea-hypopnea index (AHI) and oxygen desaturation index (ODI) values are also summarized in Table 1.

Functional Head Impulse Test (fHIT) analysis revealed vestibular impairment in 6 out of 20 patients (30.0%).

Following the completion of the reHAB therapy protocol, repeat polysomnography (PSG) demonstrated changes in OSA severity: 5 patients (25.0%) had mild OSA, 14 patients (70.0%) had moderate OSA, and only 1 patient (5.0%) remained in the severe OSA category (Table 1).

Summary statistics for the 20 patients, reporting Age, BMI, and pre-/post-treatment AHI and ODI as mean (SD) and median (IQR) are presented in Table 2.

**Table 2:** Summary statistics for the 20 patients, reporting Age, BMI, and pre-/post-treatment AHI and ODI as mean (SD) and median (IQR).

**N**	20
AGE	44.1 (10.1) 46 (41; 50)
BMI	27.9 (4.9) 27 (25; 29)
AHI pre	22.6 (7.4) 22.8 (16.9; 25.6)
AHI post	18.5 (8.0) 17.4 (15.4; 22.1)
ODI pre	22 (9.1) 21.4 (15.5; 27.2)
ODI post	18.7 (9.9) 16.0 (11.9; 24.6)

**Figure 3:**
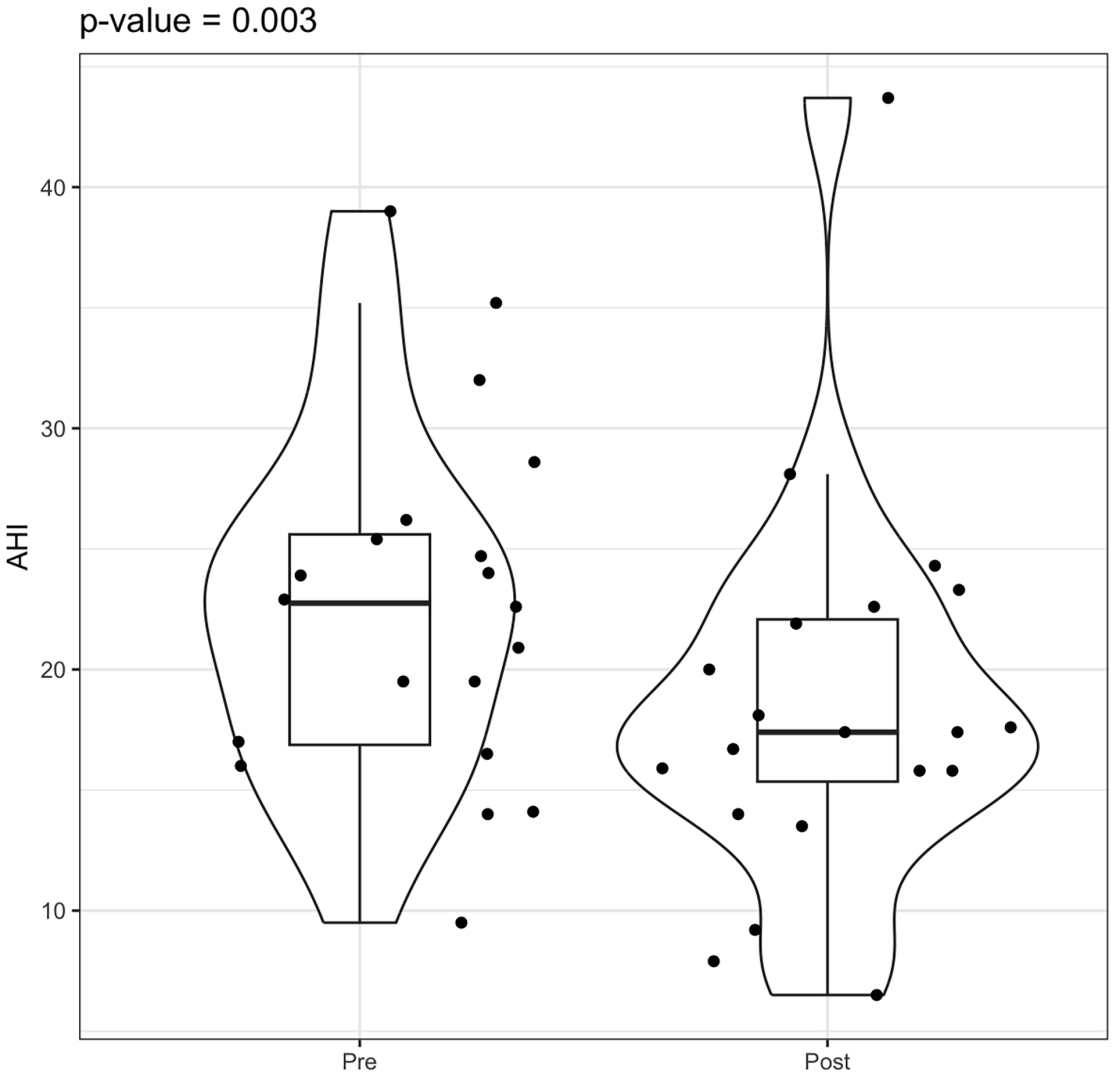
Violin box plot AHI pre/post-rehab.

**Figure 4:**
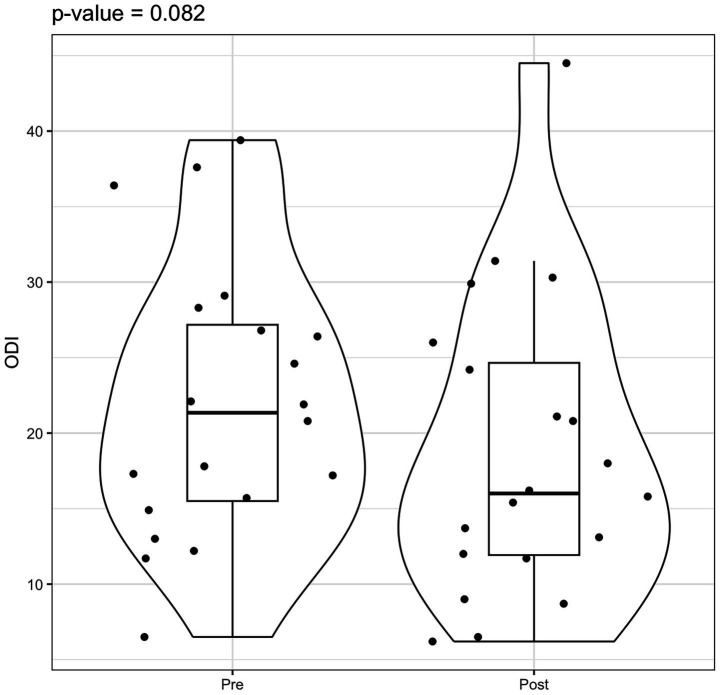
Violin box plot ODI pre/post rehab.

**Figure 5:**
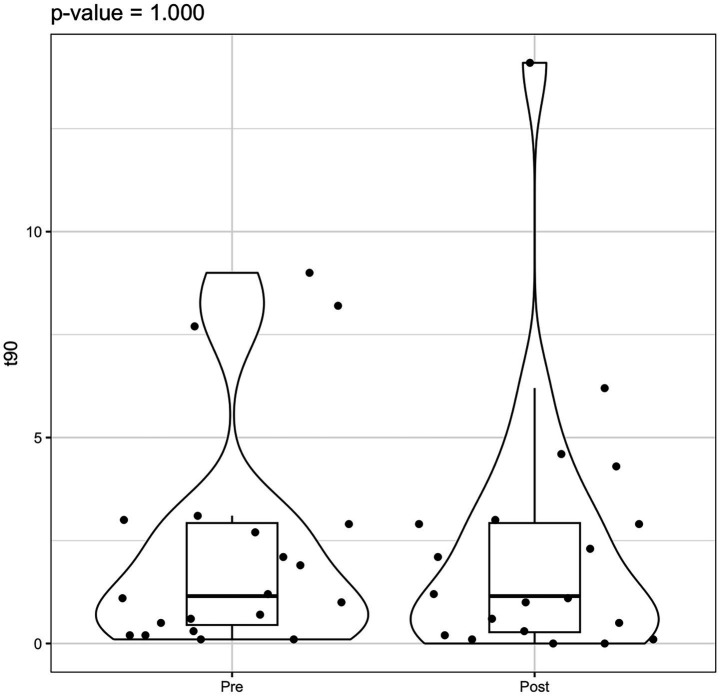
Violin box plot t90 pre/post rehab.

Statistical analysis using the Wilcoxon signed-rank test indicated a significant reduction in AHI values post-rehabilitation (*p* = 0.003). However, the change in ODI did not reach statistical significance (*p* = 0.082) (Figures 3–5).

**Figure 6:**
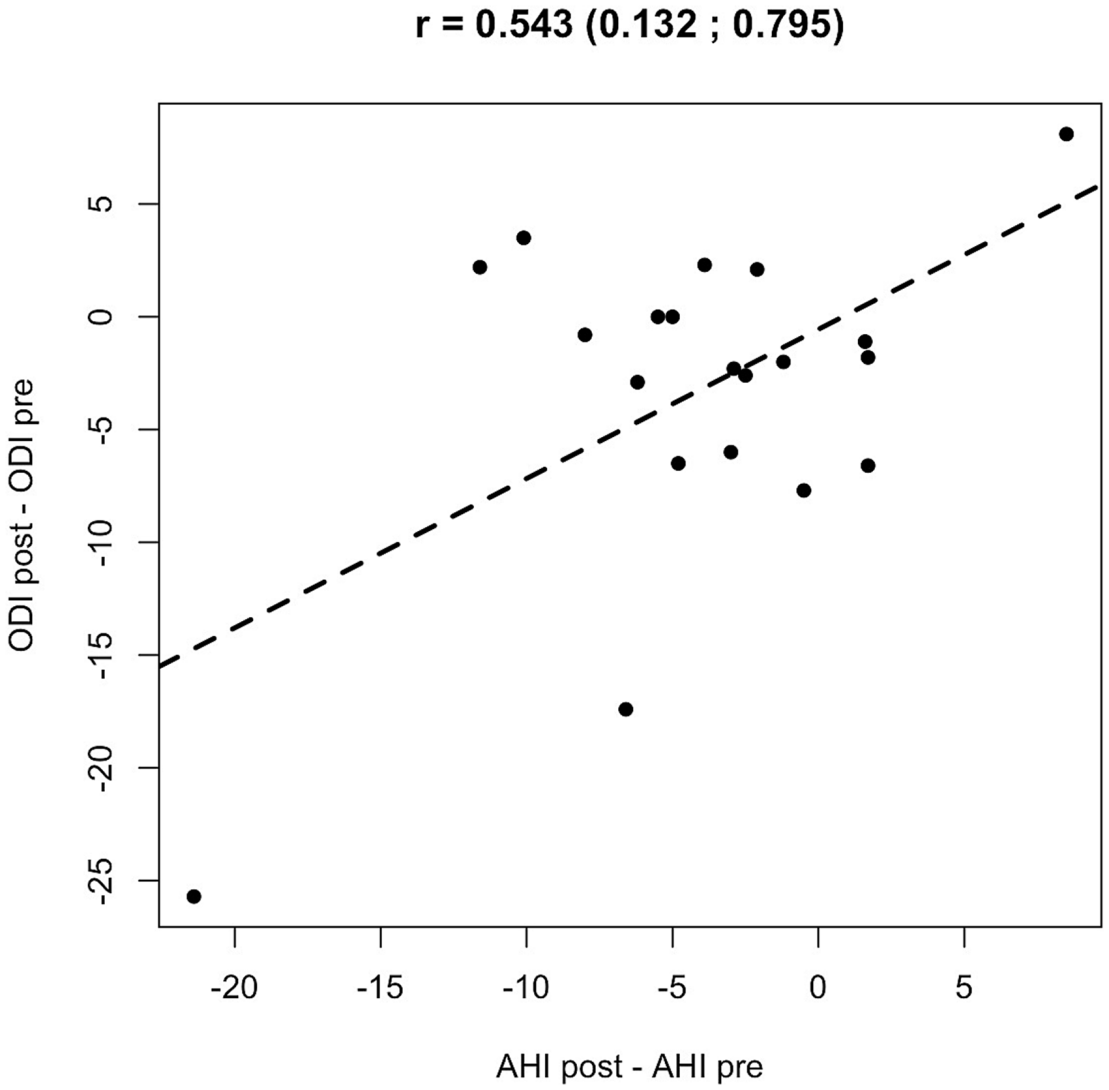
Scatter plot and Pearson’s correlation coefficient between AHI and ODI pre/post reHAB therapy.

A scatter plot analysis revealed a moderate positive correlation between the changes in AHI and ODI pre- and post-reHAB therapy (Figure 6), with a Pearson correlation coefficient of 0.543 (95% CI: 0.132–0.795). No significant correlations were found between age or BMI and changes in AHI or ODI following rehabilitation.

Regarding vestibular function, fHIT results showed improvement in all six patients with previously impaired values. Of these, two patients returned to normal vestibular function, while the remaining four continued to demonstrate impairment, albeit with improved performance metrics.

## 4 Discussion

The vestibular system is a highly integrated network responsible for detecting head position and movement in space, thereby enabling the coordination of eye movements, posture, and balance. Emerging evidence has proposed a link between the vestibular system and the neural pathways regulating sleep. Obstructive sleep apnea (OSA), a prevalent disorder characterized by intermittent nocturnal hypoxia, is associated with oxidative stress, sympathetic hyperactivity, endothelial dysfunction, and metabolic disturbances (16, 17).

The basis for this proposed association is both anatomical and physiological. Gallina et al. (5) described a potential anatomical correlation between the vestibular nuclei (VN) and structures involved in respiratory control, particularly the adjacent parabrachial nuclei. Their study, which employed polysomnography (PSG), video-oculonystagmography, and caloric testing in OSA patients, demonstrated qualitative and quantitative vestibular dysfunction, particularly in individuals with elevated AHI. The authors also hypothesized that sleep deprivation may lead to abnormal postural control and disruption of the vestibular-proprioceptive balance.

Physiological connections between the vestibular nuclei and sleep-regulating structures have been demonstrated in both animal models and human studies. These investigations have shown that the suprachiasmatic nucleus (SCN)—the central pacemaker of the circadian rhythm—communicates with the VN via the lateral geniculate nucleus (4, 6). The SCN receives cholinergic input that promotes cortical arousal and wakefulness. This pathway is further modulated by orexinergic input from hypothalamic neurons located in the perifornical region, posterior nucleus, and lateral hypothalamic area (17). Conversely, the ventrolateral preoptic nucleus (VLPO), through GABAergic signaling, counteracts this arousal pathway to facilitate sleep onset and maintenance. Disruption of the SCN–VN pathway in animal studies has led to wake-sleep disturbances resembling narcolepsy and cataplexy. Human data support this interaction as well, notably among astronauts, who frequently report sleep disturbances during microgravity exposure—presumably due to the absence of vestibular stimulation (18–22).

Based on this framework, we previously evaluated the Functional Head Impulse Test (fHIT) in a larger cohort of 85 individuals (50 OSA patients and 35 controls) (7). That study revealed vestibular impairment in 68% of OSA patients, with no clear correlation to disease severity, and demonstrated a statistically significant difference in Z-score distributions between OSA and control groups (*p* < 0.001 for all comparisons). These findings corroborated earlier work by Cavdar et al. (4). In the present study, similar but less pronounced findings were observed, with 30% of patients exhibiting vestibular impairment. This discrepancy may be attributed to the smaller sample size of the current, preliminary study.

The second objective of this study was to evaluate whether vestibular rehabilitation, through the reHAB protocol, could influence key PSG parameters—specifically AHI and ODI. Notably, despite the short duration of the intervention, significant improvements were observed in AHI, suggesting a potential therapeutic benefit of vestibular stimulation on sleep-related respiratory parameters. In contrast, ODI did not demonstrate a statistically significant change.

Notably, two participants exhibited worsening on select endpoints (e.g., increases in AHI or ODI). In a small pilot cohort, such non-response is not unexpected and may reflect night-to-night variability in OSA severity, regression to the mean, or heterogeneous pathophysiology rather than a treatment-related detriment.

To minimize confounding variables, only patients with isolated OSA were included. Furthermore, only individuals with a Dizziness Handicap Inventory (DHI) score below 40 (non-pathological) were enrolled to assess subclinical vestibular deficits. Interestingly, both patients with normal and impaired fHIT values demonstrated functional improvements following reHAB therapy, with 90% of the cohort showing benefit. The sole patient who did not exhibit improvement had the highest BMI (39 kg/m^2^), suggesting that greater adiposity may attenuate the effects of vestibular rehabilitation or that a longer duration of therapy may be required to elicit a measurable benefit in such cases.

These preliminary findings support the hypothesis of a functional connection between the vestibular nuclei and the neural pathways regulating sleep, as evidenced by the observed improvements in both vestibular function and apnea-hypopnea index (AHI) following vestibular rehabilitation. Vestibular stimulation may enhance the activity of the parabrachial nuclei and indirectly modulate the orexinergic system, contributing to improved sleep regulation in patients with obstructive sleep apnea (OSA).

However, as a pilot study, this work has certain limitations, including the small sample size and the absence of a comprehensive evaluation of the posterior labyrinth, particularly due to the lack of vestibular evoked myogenic potential (VEMP) testing (17–19). The study was conceived as an initial exploratory analysis to examine the relationship between OSA and vestibular function. Ongoing research aims to conduct a more extensive analysis of the vestibular system—both before and after reHAB therapy—to validate and expand upon these initial results.

## Data Availability

The original contributions presented in the study are included in the article/supplementary material, further inquiries can be directed to the corresponding author.
